# Machine learning models for prediction of adverse events after percutaneous coronary intervention

**DOI:** 10.1038/s41598-022-10346-1

**Published:** 2022-04-15

**Authors:** Nozomi Niimi, Yasuyuki Shiraishi, Mitsuaki Sawano, Nobuhiro Ikemura, Taku Inohara, Ikuko Ueda, Keiichi Fukuda, Shun Kohsaka

**Affiliations:** 1 Department of Cardiology, Keio University School of Medicine, 35 Shinanomachi, Shinjuku-ku, Tokyo, Japan; 2 Department of Cardiology, Tokyo Dental College Ichikawa General Hospital, Chiba, Japan

**Keywords:** Cardiology, Interventional cardiology

## Abstract

An accurate prediction of major adverse events after percutaneous coronary intervention (PCI) improves clinical decisions and specific interventions. To determine whether machine learning (ML) techniques predict peri-PCI adverse events [acute kidney injury (AKI), bleeding, and in-hospital mortality] with better discrimination or calibration than the National Cardiovascular Data Registry (NCDR-CathPCI) risk scores, we developed logistic regression and gradient descent boosting (XGBoost) models for each outcome using data from a prospective, all-comer, multicenter registry that enrolled consecutive coronary artery disease patients undergoing PCI in Japan between 2008 and 2020. The NCDR-CathPCI risk scores demonstrated good discrimination for each outcome (C-statistics of 0.82, 0.76, and 0.95 for AKI, bleeding, and in-hospital mortality) with considerable calibration. Compared with the NCDR-CathPCI risk scores, the XGBoost models modestly improved discrimination for AKI and bleeding (C-statistics of 0.84 in AKI, and 0.79 in bleeding) but not for in-hospital mortality (C-statistics of 0.96). The calibration plot demonstrated that the XGBoost model overestimated the risk for in-hospital mortality in low-risk patients. All of the original NCDR-CathPCI risk scores for adverse periprocedural events showed adequate discrimination and calibration within our cohort. When using the ML-based technique, however, the improvement in the overall risk prediction was minimal.

## Introduction

Percutaneous coronary intervention (PCI) for patients with coronary artery disease (CAD) has become widely performed^1^. While advances in devices and treatment strategies, residual risks of periprocedural adverse events such as acute kidney injury (AKI), bleeding, and death, remain^2,3^. Therefore, accurate and easy-to-use risk stratification tools for estimating the risk of these complications can provide a basis for shared decision-making and specific interventions such as bleeding avoidance strategies. For example, The United States National Cardiovascular Data Registry (NCDR) has developed risk scores (NCDR-CathPCI risk score) for periprocedural adverse events using a traditional logistic regression (LR) model with approximately 10 routinely collected preprocedural variables^4–6^, and they have been widely validated among different regions and races^7^.

Machine learning (ML) techniques have recently become a promising alternative approach for clinical decision support, especially in non-structured highly complex data. In fact, the number of publications focusing on ML in cardiology research has been increasing (up to 1 out of every 1,000 new publications in 2020), and the United States Food and Drug Administration has already approved a number of ML products for use in cardiology^8^. However, when using data from the structured electronic health record, whether the ML models improve the prediction performance of adverse periprocedural events compared to the classical LR model such as NCDR-CathPCI risk scores remains unknown. The aims of this study were (1) to evaluate the performance of the NCDR-CathPCI models in Japanese patients with CAD who underwent PCI, (2) to develop LR based and modern ML-based models using the same variables as the NCDR-CathPCI models, and (3) to compare the individual performances of the original NCDR-CathPCI, LR-based, and ML-based models.

## Methods

### Data source

The Japan Cardiovascular Database-Keio Interhospital Cardiovascular Studies (JCD-KiCS) is a large, ongoing, prospective multicenter (n = 15) PCI registry to collect clinical data of consecutive patients undergoing PCI in Japan that developed in collaboration with the National Cardiovascular Data Registry (NCDR) CathPCI^9–11^. In JCD-KiCS, all PCI procedures were conducted under the direction of the intervention team of each participating hospital according to standard care. Participating hospitals were instructed to register data from consecutive PCI using an electronic data-capturing software system equipped with a data query engine and validations to maintain data quality. Data entry was conducted by dedicated clinical research coordinators who trained for JCD-KiCS specifically. Data quality was ensured through the use of an automatic validation system and bimonthly standardized education and training for the clinical research coordinators. The senior study coordinator (I.U.) and extensive on-site auditing by the investigator (S.K.) ensured proper registration of each patient. The protocol of this study was under the principles of the Declaration of Helsinki and approved by the Keio University School of Medicine Ethics Committee and the committee of each participating hospital (National Hospital Organization Review Board for Clinical Trials; the Eiju General Hospital Ethics Committee; the Ethics Committee of Saiseikai Utsunomiya Hospital; the Research Ethics Committee, Tokyo Saiseikai Central Hospital; the Japanese Red Cross Ashikaga Hospital Ethics Committee; Kawasaki Municipal Hospital Institutional Review Board; Saitama City Hospital Ethical Review Board; Isehara Kyodo Hospital Institutional Review Board; Tokyo Dental College Ichikawa General Hospital Institutional Review Board; the Independent Ethics Committee of Hiratsuka City Hospital; The Saint Luke’s Health System Institutional Review Board; the Hino Municipal Hospital Institutional Review Board; and the Ethics Committee of Yokohama Municipal Citizen’s Hospital). All participants were provided verbal or written consent for the baseline data collection, and informed consent was obtained from all participants individually.

### Study population

**Figure 1:**
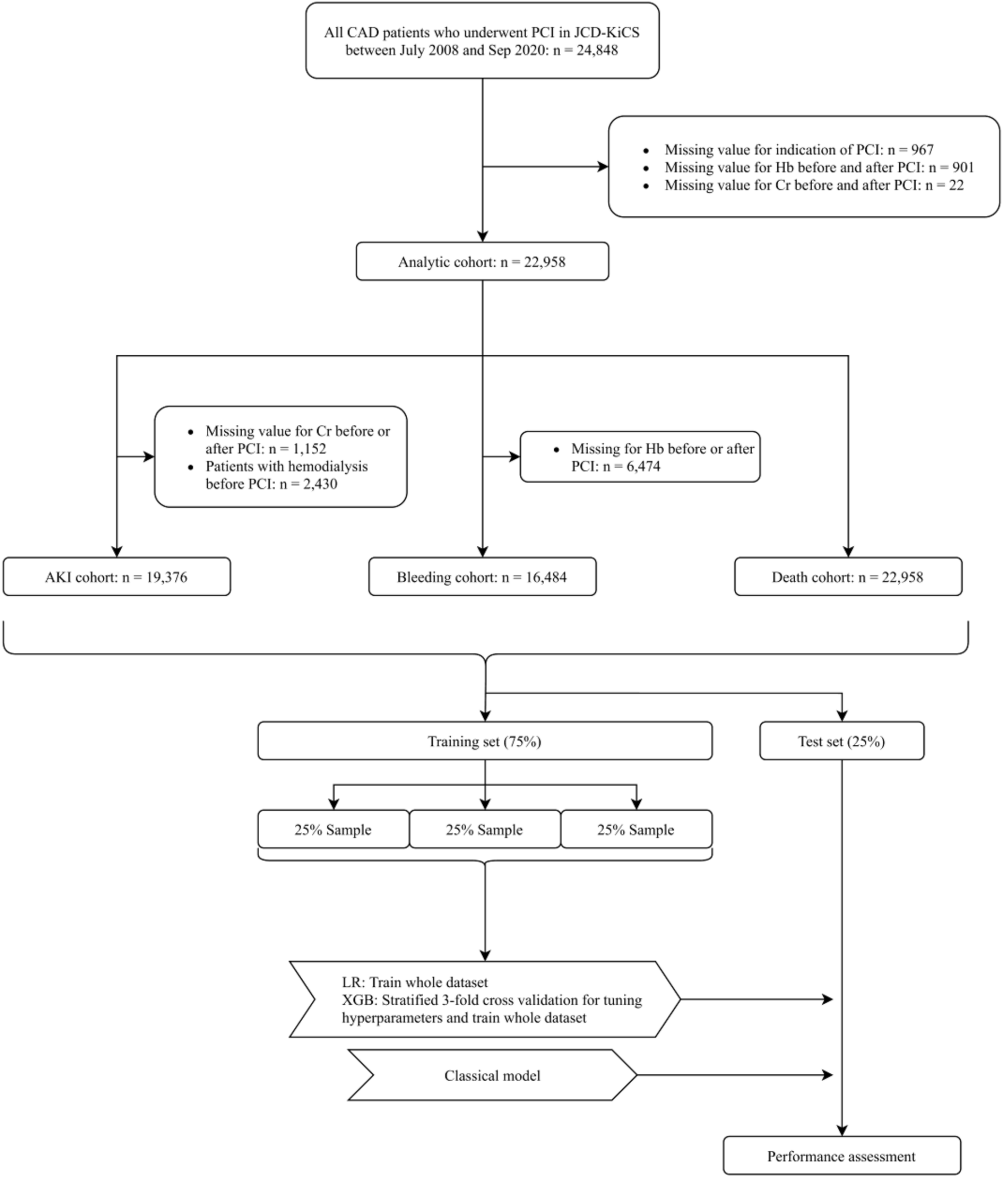
Study flowchart. Abbreviations: CAD, coronary artery disease; PCI, percutaneous coronary intervention; JCD-KiCS, The Japan Cardiovascular Database-Keio Interhospital Cardiovascular Studies; Hb, aemoglobin; Cr, creatinine; AKI, acute kidney injury; LR logistic regression model; XGB, extreme gradient boosting model.

We extracted 24,848 consecutive patients who underwent PCI between July 2008 and September 2020. Because several parameters are applied as input variables for one model and the exclusion criteria of other models (e.g., hemodialysis before PCI is an input variable of the in-hospital mortality model and exclusion criteria of the AKI model), we made each outcome-specific cohort using a two-step procedure. First, we excluded patients with missing indications (n = 967), those without pre- and post-procedure hemoglobin (n = 901), and those without pre- and post-procedure serum creatinine (n = 22) (analytic cohort). Next, we applied outcome-specific exclusion criteria, followed by the imputation of missing values to make each cohort (detailed in Fig. 1). Each population was randomly split into a training set of 75% of the patients and a test set of the remaining 25% of the patients with approximately the same proportion of events.

### Definitions and outcomes

The definition of AKI, bleeding, and in-hospital mortality were consistent with original NCDR-CathPCI models^4–6^. Briefly, AKI was defined as a ≥ 0.3 mg/dl absolute or as a ≥ 1.5-fold relative increase in post-PCI creatinine or new dialysis initiation. Bleeding was defined as any of the following occurring within 72 h after PCI or before hospital discharge (whichever occurs first): site-reported arterial access site bleeding; retroperitoneal, gastrointestinal, genitourinary bleeding, intracranial hemorrhage, cardiac tamponade, or post-procedure hemoglobin decrease of 3 g/dl in patients with pre-procedure haemoglobin ≤ 16 g/dl, or post-procedure non-bypass surgery-related blood transfusion for patients with a pre-procedure haemoglobin ≥ 8 g/dl. In-hospital mortality was defined as any post-procedural death at the same hospital admission. Because JCD-KiCS was developed in collaboration with NCDR-Cath PCI, the majority of clinical variables were defined in accord with the data dictionary (version 4.1)^9^. For example, cardiogenic shock was defined as a sustained (> 30 min) episode of systolic blood pressure of < 90 mm Hg, and/or cardiac index of < 2.2 L/min/m^2^ determined to be secondary to cardiac dysfunction, and/or the requirement for intravenous inotropic or vasopressor agents or mechanical support to maintain the blood pressure and cardiac index above the specified levels within 24 h after the procedure.

### Handling missing data

After enrollment of the analytic cohort, we imputed the missing value of pre-procedural hemoglobin with the value of post-procedural hemoglobin for the developed AKI and in-hospital mortality model, and imputed missing values of pre-procedural creatinine with those of post-procedural creatinine for the developed bleeding and in-hospital mortality models. Given that the absence rate was < 5% for any other variables, we handled the missing values to use a median imputation for the continuous variables and mode imputation for the categorical variables.

### Model development

We developed two models: LR models and extreme gradient descent boosting (XGB) models. XGB is an ML algorithm that creates a series of relatively simple decision trees combined with boosting methods to develop more robust final predictions. In the LR model, we used the same categorized variables of the original NCDR-CathPCI risk scores (original model), and in the XGB model, we used the same variables but treated raw continuous variables that were categorized in the original models. The full list of variables was as follows:

1. AKI model: age (categorized as < 50, 50–59, 60–69, 70–79, 80–89, and ≥ 90 years), heart failure within 2 weeks, estimated glomerular filtration rate (eGFR) (categorized as < 30, 30–44, 45–59, and ≥ 60 ml/min/1.73 m^2^), diabetes mellitus, prior heart failure, prior cerebrovascular disease, non ST-elevation acute coronary syndrome (NSTEACS), ST-elevation myocardial infarction (STEMI), cardiogenic shock at presentation, cardiopulmonary arrest at presentation, anemia defined as hemoglobin at admission of less than 10 g/dL, and use of IABP.
2. Bleeding model: STEMI, age (categorized as < 60, 60–70, 71–79, and ≥ 80 years), BMI (categorized as < 20, 20–30, 30–39, and ≥ 40 kg/m^2^), prior PCI, eGFR (categorized as < 30, 30–44, 45–59, and ≥ 60 ml/min/1.73 m^2^), cardiogenic shock at presentation, female sex, hemoglobin at presentation (categorized as < 13, 13–15, ≥ 15 g/dL), and PCI status (Emergency, Salvage, Urgency, and Elective).
3. In-hospital mortality model: age (categorized as < 60, 60–69, 70–79, and ≥ 80 years), cardiogenic shock at presentation, prior heart failure, peripheral artery disease, chronic obstructive pulmonary disease, estimated GFR (categorized as < 30, 30–44, 45–59, 60–89, and ≥ 90 ml/min/1.73 m^2^), NYHA classification IV at presentation, STEMI, and PCI status (emergency, salvage, urgency, and elective).

To optimize the hyperparameters of the XGB model, we used a stratified threefold cross-validation with a random search. After determining the best hyperparameters, XGB models were developed using the entire training set (hold-out methods, Supplementary Material for a more detailed explanation). In addition, we constructed the expanded LR and XGB models using additional variables selected by clinical significance. The additional variables were as follows:

- **Expanded AKI model**: contrast volume and timing of PCI (i.e., during working or holiday times).
- **Expanded bleeding model**: number of antiplatelet agents, use of anticoagulants at PCI, and timing of PCI.
- **Expanded in-hospital mortality model**: technical failure of PCI, defined as failure to cross the guidewires or when the TIMI grade after PCI was 1 or 0 (slow flow or no flow), and the timing of PCI.

### Statistics and key metrics

Continuous variables were summarized as medians with interquartile ranges and compared using Mann–Whitney U tests, and categorical variables were summarized as frequencies and compared using chi-square tests or Fisher’s exact tests, as appropriate.

The C-statistics with 95% confidence intervals (95%CIs) based on the Delong method and the area under the precision-recall area under curve (PRAUC) were used to estimate the model discrimination. Model calibration was assessed using the Brier score and calibration plot. The Brier score is defined as the mean squared difference between the observed and predicted outcomes and ranges from 0 to 1.00, with 0 representing the best possible calibration. The two primary components decomposed from the Brier score, i.e., reliability and resolution, were also evaluated. Calibration plots were used to plot the mean risk score relative to the observed outcome rate for a given quintile of the predicted risk. Furthermore, we used the net reclassification index (NRI) to evaluate the clinical utility of the LR and XGB models with cut-off values of 10%, 4%, and 2.5% for AKI, bleeding, and in-hospital mortality, respectively. A *P* value of < 0.05 was considered statistically significant. This study is based on the transparent reporting of a multivariable prediction model for individual prognosis or diagnosis (TRIPOD) guidelines.

### Sensitivity analysis

We used a multiple imputation method to handle missing values instead of a median imputation method. The multiple imputation model included all prespecified predictors and outcomes as recommended^12^. Ten imputed datasets were generated, and the C-statistics were combined using Rubin’s rules.

### Software Implementation

All analyses were conducted in R (version 4.0.4; R Project for Statistical Computing, Vienna, Austria) with tidymodels (version 0.1.2) bundle of packages for data pre-processing, hyperparameter tuning, learning, and performance metrics^13–15^. We used xgboost (version 1.3.2.1) for extreme gradient descent boosting^16^, pROC (version 1.17.0.1) to calculating C-statistics^17^, verification (version 1.42) to calculate Brier scores^18^, predictABEL (version 1.2.4) to calculate the NRI^19^ mice (version 3.14.0) to perform multiple imputation^20^.

## Results

### Patient characteristics

Between July 2008 and September 2020, a total of 22,958 consecutive patients with CAD who underwent PCI were analyzed. The patients were predominantly men with a median age of 70 (interquartile range [IQR] 62, 77) years, and a body mass index of 24.0 (21.9, 26.3). Overall, 55.4% of the patients had stable ischemic heart disease, and 58.6% underwent elective PCI. The prevalence of AKI, bleeding, and in-hospital mortality were 9.6%, 7.8%, and 2.3%, respectively (Table 1). The baseline characteristics of patients in training and test set of each outcome were in Table S1 from Supplementary Material.

**Table 1:** Baseline characteristics in analytic cohort.

Clinical Characteristics	N = 22,958
Age (years)	70 (62, 77)
Male (%)	18,213 (79.3%)
BMI (kg/m^2^)	24.0 (21.9, 26.3)
Diabetes mellitus (%)	9985 (43.5%)
Ejection fraction (%)	60 (50, 68)
PAD (%)	2118 (9.2%)
COPD (%)	749 (3.3%)
Past history of MI (%)	5466 (23.8%)
Past history of HF (%)	2228 (9.7%)
eGFR (ml/min/1.73 m^2^)	62 (48, 74)
Hb before PCI (g/dL)	13.3 (11.8, 14.6)
Hemodialysis (%)	1152 (5.0%)
**Indication (%)**	
STEMI	5083 (22.1%)
NSTEACS	5163 (22.5%)
SIHD	12,712 (55.4%)
**Urgency (%)**	
Salvage	379 (1.7%)
Emergent	4893 (21.3%)
Urgent	4225 (18.4%)
Elective	13,461 (58.6%)
AKI (%)	2194 (9.6%)
Bleeding (%)	1784 (7.8%)
In-hospital death (%)	529 (2.3%)

Data presented as median [interquartile range (IQR)] or n (%).

*BMI* body mass index, *PAD* peripheral artery disease, *COPD* chronic obstructive pulmonary disease, *MI* myocardial infarction, *HF* heart failure, *eGFR* estimated glomerular filtration rate, *Hb* hemoglobin, *STEMI* ST-elevation myocardial infarction, *NSTEACS* non ST-elevation acute coronary syndrome, *SIHD* stable ischemic heart disease, *AKI* acute kidney disease.

### Model discrimination

**Figure 2:**
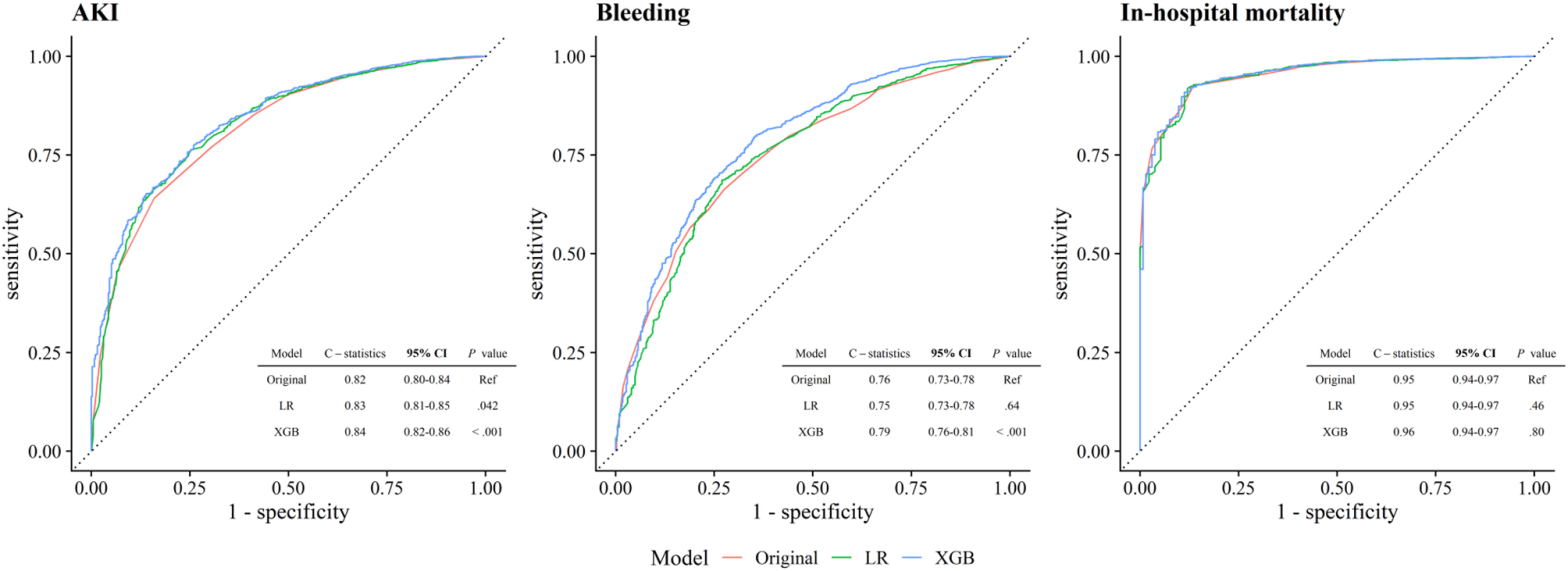
Receiver Operating Characteristic Curves for AKI, Bleeding, and In-hospital Mortality in The Test Cohort. Abbreviations: AKI, acute kidney injury; LR, logistic regression model; XGB, XGB, extreme gradient boosting model; CI, confidence interval; Ref, reference.

The original models for each outcome showed good discrimination (C-statistics of 0.82, 95% CI [0.80–0.84] for AKI; C-statistics of 0.76, 95% CI [0.73–0.78] for bleeding; C-statistics of 0.95, 95% CI [0.94–0.97] for in-hospital mortality). The LR model modestly improved the discrimination in AKI (C-statistics of 0.83, 95% CI [0.81–0.85], *P* = 0.04). The XGB models also modestly improve the discrimination in AKI and bleeding (C-statistics of 0.84, 95% CI [0.82–0.86], *P* < 0.001 for AKI; C-statistics of 0.79, 95% CI [0.76–0.81], *P* < 0.001 for bleeding) but not in-hospital mortality (Fig. 2). The performance of each model, including PRAUC, was presented in Table 2. Further, the expanded models did not improve discrimination over the original models (Table S3). Using a multiple imputation dataset, the main results were consistent with the main findings (Table S4).

**Table 2:** Performance characteristics of models for each outcome.

Characteristics	AKI			Bleeding			In-hospital mortality		
	Original	LR	XGB	Original	LR	XGB	Original	LR	XGB
Precision-recall AUC	0.351	0.347	0.363	0.262	0.289	0.393	0.377	0.378	0.400
C-statistics	0.818	0.827	0.838	0.755	0.753	0.788	0.954	0.952	0.955
Brier, total	0.064	0.064	0.062	0.087	0.084	0.081	0.021	0.019	0.019
Brier, resolution	0.0097	0.011	0.012	0.0076	0.0095	0.015	0.0051	0.0055	0.0054
Brier, reliability	0.0005	0.0006	0.0004	0.0012	0.0004	0.0003	0.0035	0.0023	0.0018

*AUC* area under curve, *AKI* acute kidney disease, *LR* logistic regression model, *XGB* extreme gradient boosting model.

### Model calibration

**Figure 3:**
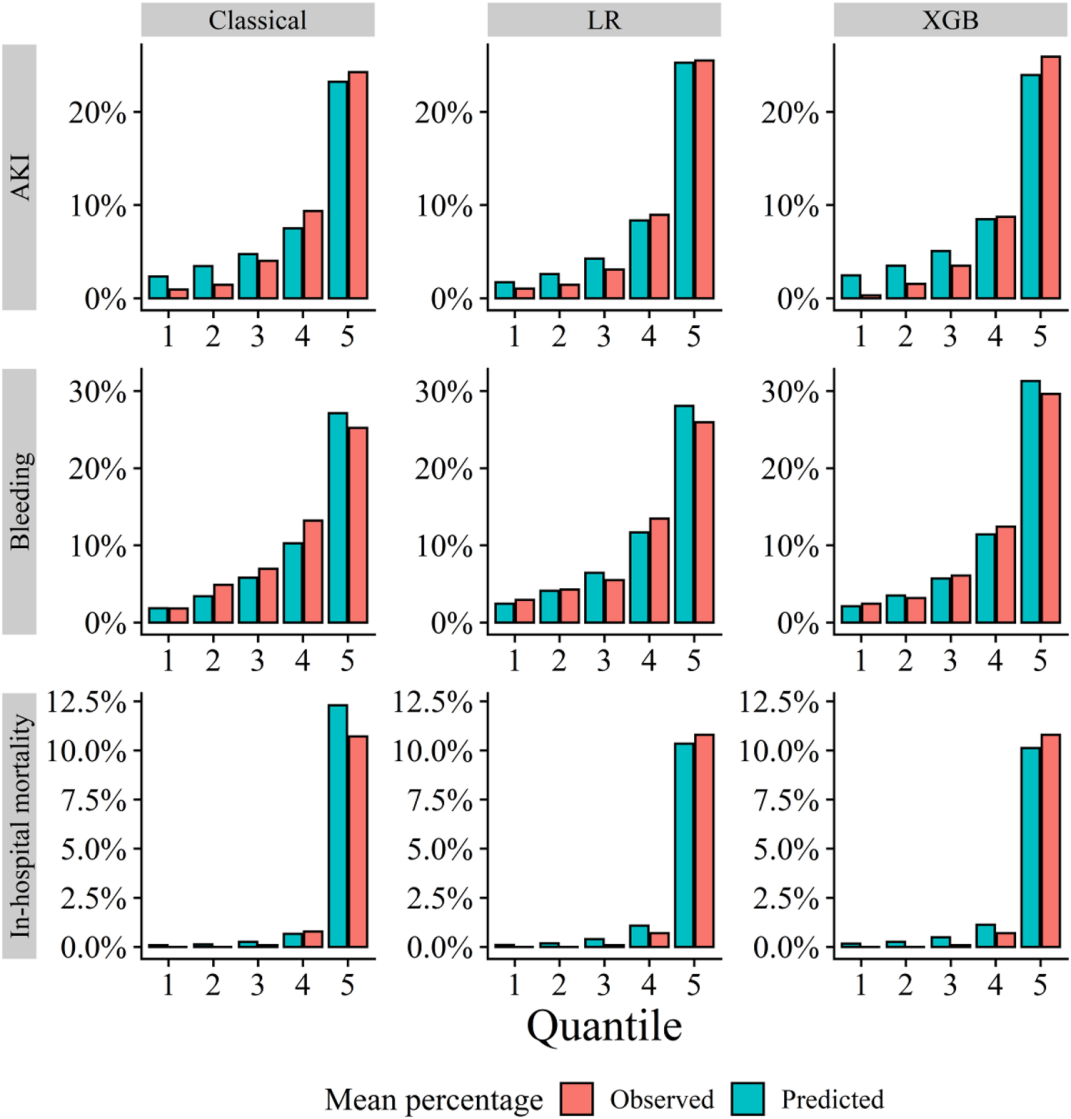
Risk of Observed AKI, Bleeding, and In-hospital mortality According to Quantiles of Event Probability Based on Each Model. AKI, acute kidney injury; LR, logistic regression model; XGB, extreme gradient boosting model.

In the original models, the calibration was adequate for each outcome (Brier score of 0.064 for AKI, 0.087 for bleeding, and 0.021 for in-hospital mortality). Whereas XGB models and LR models showed equivalent to the original models for each outcome in the Brier score and its components, the calibration plot showed an overestimated in-hospital mortality in low-risk patients (Fig. 3). The patients in the first and second quintile of the XGB model were likely to be elective cases with SIHD for PCI indication, and no patient presented with cardiogenic shock. Notably, there were no in-hospital deaths among these low-risk patients. The discrimination and calibration of the original models for the total cohort are shown in Table S2.

### Model reclassification

Compared with the original models, the LR models improved the reclassification for AKI, whereas no difference was observed in the bleeding, and a decline in the net reclassification index was shown in the in-hospital mortality. The XGB models improved the reclassification of AKI and bleeding but declined the reclassification for in-hospital death (Table 3).

**Table 3:** Net Reclassification Indices (NRIs) for machine learning models compared to original models.

Outcome	Model	NRI	95% CI	*P* value
AKI	LR	0.64	0.55, 0.74	< .001
	XGB	0.65	0.55, 0.74	< .001
Bleeding	LR	−0.03	−0.13, 0.07	.54
	XGB	0.18	0.09, 0.28	< .001
In-hospital mortality	LR	−0.69	−0.85, −0.52	< .001
	XGB	−0.93	−1.09, −0.76	< .001

*AKI* acute kidney injury, *LR* logistic regression model, *XGB* extreme gradient boosting model, *NRI* net reclassified index, *CI* confidence interval.

## Discussion

Using a Japanese multicenter PCI registry that was constructed in-sync with NCDR, we demonstrated: (1) The original NCDR CathPCI risk scores for predicting the incidence of each outcome showed a considerable performance in terms of the discrimination and calibration in Japan, and (2) compared with the original NCDR-CathPCI risk scores, ML models showed no or modest improvement in the discrimination and decreased calibration, particularly in-hospital mortality.

In our analysis, the C-statistics of all NCDR-CathPCI risk scores were more than 0.75, which was considered clearly useful discrimination^21^. While the discrimination of the ML models being better than that of the original models with a statistical significance, the absolute difference in C-statistics was minimal (0.02 in AKI and bleeding). In addition, while a sufficient calibration performance is necessary to apply in clinical practice^21^, the XGB model of in-hospital mortality was overestimated in patients in the low-risk category. This falsely high mortality risk may lead a patient to choose not to undergo a procedure inappropriately. Such poor calibration in ML models related to LR models is consistent with a previous study^22^. The plausible mechanism of overestimation in the low-risk category in in-hospital mortality might be largely owed to the low event rates observed in this group; there were no in-hospital deaths among the low-category patients. Imbalanced data pose a challenge in the machine learning field. A previous study showed that calibration performance in imbalanced data is biased because ML-based models considered the majority class to be more important than the minority class^23^. Furthermore, we constructed machine learning models based on the best AUROC values. This metric was known to be less sensitive to imbalanced data, and PRUAC was the preferred metric when data was imbalanced^24^. While AUROC has potential limitations, it was the most common metric for evaluating the prediction models and the most intuitive, whereas PRAUC did not have such a “rule of thumb^21^.” Considering the above, caution is required when constructing ML-based models using imbalanced data. Further research is needed to construct ML-based models for the imbalanced data.

ML techniques are data-driven and do not require several assumptions, whereas LR models are theory-driven and require several assumptions such as data distribution, variance equality, and linearity. Owing to freedom from these assumptions, ML models can handle non-linearity associations and interactions naturally^25^. Therefore, ML models are useful when the outcome and input variables have a complex relationship. A previous study showed that a gradient boosting model with age, sex, and paired high-sensitivity cardiac troponin-I (hs-TnI) showed better performance in predicting myocardial infarction (AUROC of 0.963 [0.956–0.971] in early and late presentation) than the ESC 0/3 h pathway^26^. ML techniques, such as deep neural networking algorithms, have shown excellent performance when dealing with high-dimensional, highly self-correlated data such as medical imaging that could not be dealt with classic statistical models^27^. Furthermore, the ML technique can recognize negligible change that humans cannot in time-dependent continuous variables, such as in electrocardiograms. Indeed, the ML technique can identify the reduced ejection fraction or hypertrophic cardiomyopathy^27,28^.

Otherwise, when dealing with fewer weakly correlated clinical variables such as structured electronic health records, LR models are likely to perform as well as ML models^29^. A systematic review showed no difference in discrimination between ML-based and LR-based models when using research with a low risk of bias^30^.

Beyond the simple measurement of performance, it is important to account for the deployment and maintenance of risk models. Both of them are difficult in ML-based models due to their insufficiency of explainability and risk for overfitting^31^, whereas LR models such as the NCDR-risk scores could easily implement and update. For example, pre/post-implementation studies have shown that integrating a stratification by the NCDR-CathPCI bleeding model and using a bleeding avoidance strategy can reduce periprocedural bleeding^32^. Further, NCDR-risk scores have been updated when concerns are raised^5,33,34^. Considering the above, it would be difficult to justify using ML-based models instead of NCDR-CathPCI risk scores within our cohort. Future analyses are needed to determine whether LR or ML-based models are better for specific data structures and outcomes.

There are several limitations to this study. First, we did not conduct an external validation for the LR and XGB models. However, we first separated the test sets to avoid leakage as recommended^35^, and were no registries that collaborated with NCDR-CathPCI in Japan except for JCD-KiCS. Second, we did not modify input variables. The input variables in original risk scores were selected based on the correlation and backward elimination methods using a logistic regression model. Otherwise, XGB models can use an embedded feature selection using variable importance^36^. XGB models with the other variables may improve the performance. However, the variables we used were clinically acceptable and intuitive. Finally, we did not develop other ML models, such as support vector machines and neural networks. However, previous studies have shown that the XGB algorithm performs better than those algorithms in cardiology research^37^.

## Conclusion

All of the original NCDR-CathPCI risk scores for adverse periprocedural events showed adequate discrimination and calibration within our cohort. The discrimination of bleeding and AKI risk improved modestly when ML-based models were incorporated; however, the improvement in the overall risk prediction was minimal.

## Supplementary Information

Supplementary Information.

## Data Availability

The datasets generated during and/or analyzed during the current study are available from the corresponding author on reasonable request.
