# Supplementary material for: Machine learning models for prediction of adverse events after percutaneous coronary intervention
Source: Sci Rep. 2022 Apr 15;12:6262. doi: 10.1038/s41598-022-10346-1 (PMC9012739; doi:10.1038/s41598-022-10346-1)
Supplement: Supplementary file 1 — Supplementary Information. [file 41598_2022_10346_MOESM1_ESM.docx]

Machine Learning Models for Prediction of Adverse Events after Percutaneous Coronary Intervention

Author: Nozomi Niimi^1^, Yasuyuki Shiraishi^1^, Mitsuaki Sawano^2^, Nobuhiro Ikemura^1^, Taku Inohara^1^, Ikuko Ueda^1^, Keiichi Fukuda^1^, MD; Shun Kohsaka^1*^

1: Department of Cardiology, Keio University School of Medicine, Tokyo, Japan
2: Department of Cardiology, Tokyo Dental College Ichikawa General Hospital, Chiba, Japan

Corresponding Author: Shun Kohsaka, MD, PhD

Department of Cardiology, Keio University School of Medicine, 35 Shinanomachi, Shinjuku-ku, Tokyo, Japan

Tel: +81-3-3353-1211

Fax: +81-3-5363-3875

E-mail: [sk@keio.jp](mailto:sk@keio.jp)

# Supplemental Methods

We split randomly 75% of the data for training, and holding out 25% for validation with keeping consistent event rate. To avoid data leakage, we ran imputation techniques for missing value of both train and test dataset based on the 75% training data. Because we only use approximately 10 variables based on NCDR-CathPCI models, we did not use any regularization methods for logistic regression model (e.g., LASSO or Ridge). Therefore, we did not tune the hyperparameters for LR models. For XGB models, A random grid search was constructed across several hyperparameter combinations and looped through each combination.

The hyperparameters and their search spaces were:1) depth of tree (max_depth in xgboost packages; from 1 to 15), 2) feature subset by tree (colsample_bytree; from 1 to using all features in each model), 3) learning rate (eta; from 10-10 to 10-1), 4) subsampling rate (subsample; from 0.5 to 1.0), 5) minimum number of data points in a node that is required for the node to be split further. (min_child_weight; from 2 to 40). We made 300 hyperparameter combinations by random search methods and looped through each combination. To avoid overfitting, we used early stopping methods to determine the number of trees. We firstly set adequate trees (1,000). Next, learning was stopped if no improve within 10 iterations in test set. The hyperparameters were as follows.

| model | colsample_bynode | min_child_weight | max_depth | eta | subsample |
| --- | --- | --- | --- | --- | --- |
| AKI | 4 | 27 | 15 | 0.005169 | 0.93622472 |
| Bleeding | 9 | 38 | 4 | 0.010745 | 0.86370291 |
| In-hospital mortality | 3 | 21 | 3 | 0.008608 | 0.90280491 |

Supplementary Table S1. Baseline Characteristics in Each Dataset

|  | **AKI** | | | **Bleeding** | | | **In-hospital mortality** | | |
| --- | --- | --- | --- | --- | --- | --- | --- | --- | --- |
| **Characteristic** | **Training set,**  **N = 14,543** | **Test set,**  **N = 4,833** | ***P* value** | **Training set,**  **N = 12,409** | **Test set,**  **N = 4,075** | ***P* value** | **Training set,**  **N = 17,219** | **Test set,**  **N = 5,739** | ***P* value** |
| Age (years) | 70 (62, 77) | 70 (62, 77) | .6 | 69 (61, 77) | 70 (61, 77) | .3 | 70 (62, 77) | 70 (62, 77) | .7 |
| Male (%) | 11,498 (79.1%) | 3,844 (79.5%) | .5 | 9,719 (78.3%) | 3,232 (79.3%) | .2 | 13,642 (79.2%) | 4,571 (79.6%) | .5 |
| BMI (kg/m^2^) | 24.0 (21.9, 26.3) | 24.0 (21.9, 26.3) | .5 | 23.8 (21.8, 26.2) | 23.8 (21.7, 26.1) | .5 | 24.0 (21.9, 26.3) | 24.0 (21.9, 26.3) | .8 |
| Diabetes mellitus (%) | 6,033 (41.5%) | 2,072 (42.9%) | .090 | 5,172 (41.7%) | 1,742 (42.7%) | .2 | 7,456 (43.3%) | 2,529 (44.1%) | .3 |
| Ejection fraction (%) | 60 (50, 68) | 60 (50, 68) | .4 | 60 (48, 67) | 60 (48, 67) | .8 | 60 (50, 68) | 60 (50, 68) | .4 |
| PAD (%) | 1,193 (8.2%) | 422 (8.7%) | .2 | 1,054 (8.5%) | 346 (8.5%) | >0.9 | 1,571 (9.1%) | 547 (9.5%) | .4 |
| COPD (%) | 492 (3.4%) | 161 (3.3%) | .9 | 417 (3.4%) | 147 (3.6%) | .5 | 568 (3.3%) | 181 (3.2%) | .6 |
| Past history of MI (%) | 3,272 (22.5%) | 1,102 (22.8%) | .7 | 2,611 (21.0%) | 867 (21.3%) | .7 | 4,090 (23.8%) | 1,376 (24.0%) | .7 |
| Past history of HF (%) | 1,312 (9.0%) | 428 (8.9%) | .7 | 1,188 (9.6%) | 398 (9.8%) | .7 | 1,671 (9.7%) | 557 (9.7%) | >0.9 |
| eGFR (ml/min/1.73 m^2^) |  |  |  | 62 (48, 75) | 61 (46, 74) | .063 | 62 (49, 75) | 62 (48, 74) | .13 |
| Hb before PCI (g/dL) | 13.50 (12.10, 14.70) | 13.50 (12.00, 14.70) | .8 |  |  |  | 13.30 (11.80, 14.60) | 13.30 (11.80, 14.50) | .7 |
| Indication (%) |  |  | .13 |  |  | .10 |  |  | .047 |
| STEMI | 3,641 (25.0%) | 1,230 (25.5%) |  | 3,704 (29.8%) | 1,226 (30.1%) |  | 3,798 (22.1%) | 1,285 (22.4%) |  |
| NSTEACS | 3,526 (24.2%) | 1,103 (22.8%) |  | 3,466 (27.9%) | 1,070 (26.3%) |  | 3,940 (22.9%) | 1,223 (21.3%) |  |
| SIHD | 7,376 (50.7%) | 2,500 (51.7%) |  | 5,239 (42.2%) | 1,779 (43.7%) |  | 9,481 (55.1%) | 3,231 (56.3%) |  |
| Urgency (%) |  |  | .5 |  |  | .2 |  |  | .5 |
| Salvage | 255 (1.8%) | 87 (1.8%) |  | 263 (2.1%) | 89 (2.2%) |  | 284 (1.6%) | 95 (1.7%) |  |
| Emergent | 3,499 (24.1%) | 1,176 (24.3%) |  | 3,586 (28.9%) | 1,171 (28.7%) |  | 3,666 (21.3%) | 1,227 (21.4%) |  |
| Urgent | 2,913 (20.0%) | 919 (19.0%) |  | 2,863 (23.1%) | 882 (21.6%) |  | 3,208 (18.6%) | 1,017 (17.7%) |  |
| Elective | 7,876 (54.2%) | 2,651 (54.9%) |  | 5,697 (45.9%) | 1,933 (47.4%) |  | 10,061 (58.4%) | 3,400 (59.2%) |  |
| AKI (%) | 1,174 (8.1%) | 431 (8.9%) | .065 |  |  |  |  |  |  |
| Bleeding (%) |  |  |  | 1,356 (10.9%) | 428 (10.5%) | .4 |  |  |  |
| In-hospital mortality (%) |  |  |  |  |  |  | 387 (2.2%) | 142 (2.5%) | .3 |
| Data presented as median [interquartile range (IQR)] or n (%). Abbreviations: BMI, body mass index; PAD, peripheral artery disease; COPD, chronic obstructive pulmonary disease; MI, myocardial infarction; HF, heart failure; eGFR, estimated glomerular filtration rate; Hb, hemoglobin; STEMI, ST-elevation myocardial infarction; NSTEACS, Non ST-elevation acute coronary syndrome; SIHD, stable ischemic heart disease | | | | | | | | | |

Supplementary Table S2. Performance of Original Models for Total Cohort

| **Characteristics** | **AKI** | | **Bleeding** | | **In-hospital mortality** | |
| --- | --- | --- | --- | --- | --- | --- |
|  | **Total cohort** | **Test cohort** | **Total cohort** | **Test cohort** | **Total cohort** | **Test cohort** |
| PRAUC | 0.347 | 0.351 | 0.287 | 0.286 | 0.349 | 0.332 |
| Brier, total | 0.067 | 0.071 | 0.085 | 0.083 | 0.021 | 0.022 |
| Brier, resolution | 0.0096 | 0.010 | 0.0091 | 0.0094 | 0.0045 | 0.0046 |
| Brier, reliability | 0.0002 | 0.0002 | 0.0006 | 0.0008 | 0.0032 | 0.0033 |
| C-statistics | 0.801 | 0.792 | 0.761 | 0.764 | 0.928 | 0.922 |
| Abbreviations: PRAUC, Area under the precision-recall curve; AUROC, Area under the receiver operating characteristic curve; AKI, acute kidney disease | | | | | | |

Supplementary Table S3. The discrimination of expanded models.

|  | | | | |
| --- | --- | --- | --- | --- |
| Outcome | Model | C-statistics | 95% CI | *P* value |
| AKI | Original | 0.82 | 0.80-0.84 | Ref |
|  | expanded LR | 0.83 | 0.81-0.85 | 0.005 |
|  | expanded XGB | 0.84 | 0.82-0.86 | 0.003 |
| Bleeding | Original | 0.78 | 0.74-0.83 | Ref |
|  | expanded LR | 0.79 | 0.75-0.84 | 0.28 |
|  | expanded XGB | 0.80 | 0.75-0.84 | 0.17 |
| In-hospital mortality | Original | 0.95 | 0.94-0.97 | Ref |
|  | expanded LR | 0.96 | 0.94-0.97 | 0.85 |
|  | expanded XGB | 0.95 | 0.94-0.97 | 0.80 |
| Expanded AKI model added the timing of PCI, and contrast volume. Expanded bleeding model added the timing of PCI, number of antiplatelet agents, and concomitant anticoagulants at PCI. Expanded in-hospital mortality model added the timing of PCI, and success of PCI. Abbreviations: AKI, acute kidney injury; LR, logistic regression; XGB, extreme gradient boosting; 95% CI, 95% confidence interval. | | | | |

Supplementary Table S4. The discrimination of models of multiple imputation methods.

| Table. The discrimination of models of multiple imputation methods. | | | | |
| --- | --- | --- | --- | --- |
| Outcome | Model | C-statistics | 95% CI | P value |
| AKI | Original | 0.82 | 0.80-0.84 | Ref |
|  | LR | 0.83 | 0.81-0.85 | 0.004 |
|  | XGB | 0.84 | 0.82-0.86 | 0.002 |
| Bleeding | Original | 0.75 | 0.73-0.78 | Ref |
|  | LR | 0.75 | 0.73-0.78 | 0.63 |
|  | XGB | 0.78 | 0.76-0.80 | < 0.001 |
| In-hospital mortality | Original | 0.95 | 0.94-0.97 | Ref |
|  | LR | 0.95 | 0.94-0.97 | 0.48 |
|  | XGB | 0.96 | 0.94-0.97 | 0.74 |
| The multiple imputation model included all pre-specified predictors as well as outcomes. A total of 10 imputed datasets were generated, and the C-statistics were combined using Rubin’s rules. Abbreviations: AKI, acute kidney injury; LR, logistic regression; XGB, extreme gradient boosting; 95% CI, 95% confidence interval. | | | | |
